# Comparison between lengthening over nail and conventional Ilizarov lengthening: a prospective randomized clinical study

**DOI:** 10.1007/s11751-013-0163-x

**Published:** 2013-08-02

**Authors:** Timour F. EL-Husseini, Nabil A. M. Ghaly, Mahmoud A. Mahran, Mohamed Ahmed Al Kersh, Khaled M. Emara

**Affiliations:** Ain Shams University Hospitals, 28 Tawfik City, Nasr City, Cairo, Egypt

**Keywords:** Ilizarov, Bone lengthening, Intramedullary nail, Distraction osteogenesis

## Abstract

The aim of this study is to compare lengthening over an intramedullary nail to the conventional Ilizarov method with regard to percentage length increase, external fixation index, consolidation index and incidence of complications. This is a prospective randomized controlled study. Thirty-one limbs in 28 patients were included in the study; 15 were lengthened over an intramedullary nail, and 16 limbs were lengthened conventionally. The mean duration of external fixation in the lengthening over nail group was 52.2 days compared to 180.4 days in the conventional group. There was higher incidence of complications in the conventional method group. In comparison with conventional Ilizarov lengthening, lengthening over an intramedullary nail offers a shorter period of external fixation and fewer complications overall, but there is a high incidence of deep intramedullary infection which is serious.

## Introduction

In 1905, Codivilla [1] first described leg lengthening by distraction osteogenesis. The consolidation phase for the lengthened column of bone is approximately twice as long as the distraction phase in children but is doubled in adults in whom periods of external fixation varies between 30 and 50 days per centimetre gain in length [2]. The consolidation phase is usually poorly tolerated and associated with a high incidence of complications such as pin-track infection, angulation, scarring and knee and ankle joint stiffness. In addition, a fracture of the regenerate column of bone occurs if the fixator is removed prematurely. There is potential benefit if the period of external fixation could be reduced without increasing the likelihood of such complications [3].

Lengthening over an intramedullary nail (LON) emerged to allow early fixator removal and a more comfortable consolidation period without jeopardizing the integrity of the regenerate [4, 5]. However, several authors have encountered a high rate of complications with this method such that they have abandoned this for the conventional Ilizarov method [6–8].

**Fig. 1:**
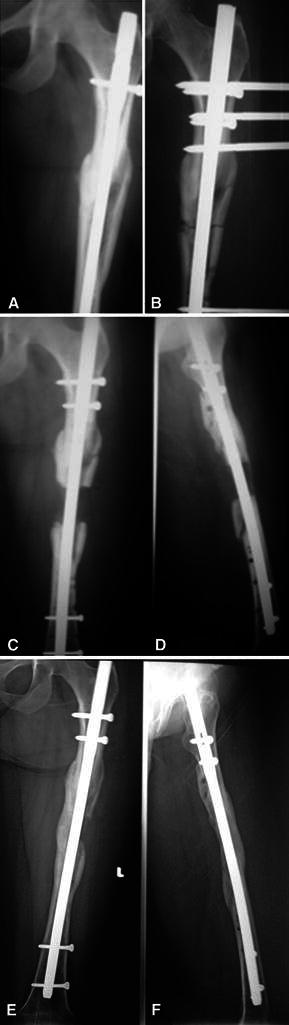
Serial X-ray of a case of lengthening over nail: **a** before lengthening. **b** After operation. **c**, **d** After removal of fixator. **e**, **f** After full consolidation

The purpose of this study is to compare LON with the conventional Ilizarov method for individuals with limb length discrepancy or short stature as regard percentage increase, external fixation index and consolidation index. To our knowledge, this is the first randomized controlled study to compare LON and lengthening by the conventional Ilizarov method (Fig. 1).

## Patients and methods

This is a prospective randomized controlled study that was conducted between July 2009 and December 2011. Thirty-one limbs in 28 individuals were lengthened. Inclusion and exclusion criteria are listed in Table 1.

**Table 1:** Inclusion and exclusion criteria

Inclusion criteria	Exclusion criteria
1. Skeletally mature patients	1. Post-osteomyelitis sequelae
2. Intramedullary canal diameter wide enough to accommodate the smallest available IMN	2. Marked deformity unsuitable for acute correction
	3. LLD <3 cm

Patients were randomized by allocating patients with an odd number to the group for LON and patients with an even number in the conventional method group. Fifteen cases with 15 short limbs had LON (9 femora and 6 tibiae), whereas 16 short limbs were lengthened conventionally (9 femora and 7 tibiae). Patients’ demographics and aetiology of LLD are shown in Table 2.

**Table 2:** Demographics and aetiology

	LON group	Conventional group
Short limbs/patients	15/15	16/13
Lengthened segments		
Femora	9	9
Tibiae	6	7
Mean age (years)	31.3	28.4
Gender (male/female)		
Males	7	6
Females	8	7
Aetiology		
Congenital	2	1
Post-traumatic	12	8
Developmental	0	4
Post-tumour resection	1	0
Type of external fixator		
LRS	6	3
Ilizarov	9	13

*LRS* limb reconstruction system

### Operative technique and postoperative protocol

The technique of LON as described by Paley et al. [4] was adopted in all patients, with some modifications. The medullary canal was reamed over an olive-tipped guide wire until a diameter 2 mm larger than that of the intended IM nail. Insertion of the IM nail was carried out with two proximal locking screws applied, and the distal locking screws were omitted, to be done in the second operation when the patient reached to the desired length. With the IM nail in place, an external fixator was then applied for lengthening.

**Fig. 2:**
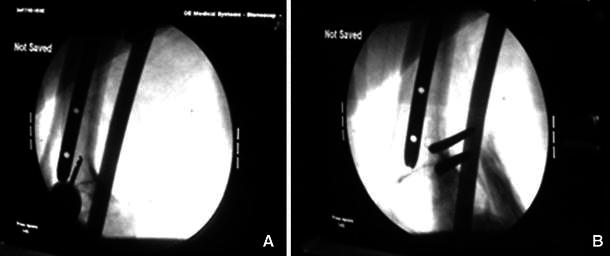
**a**, **b** Insertion of pins in a space from the nail

All external fixator pins or wires were inserted without coming in contact with the intramedullary nail. There was an approximately one millimetre or more of space between the external fixation pin and the nail. We used the image intensifier to ensure that there was space between the pins and the nail (Fig. 2).

All femoral nails were inserted in an antegrade manner. The osteotomy was done using the multiple drill-hole technique and was either in the proximal metaphysis or mid-diaphysis depending on the desired amount of lengthening.

No separate drill holes were done for venting as some venting was possible from the predrilled holes of the planned osteotomy site. This also carried the advantage of dispersing the reaming material in the vicinity of the osteotomy site.

Immediate full weight bearing with aids and a full ROM were allowed on the second postoperative day for both groups. Prophylactic intravenous antibiotics were administered for 48 h. Lengthening started on day 7–10 postoperatively at a rate of 0.25 mm four times daily. The distraction rate was modified during follow-up according to the quality of regenerate.

During distraction, patients were examined every 4 weeks and screened for local signs of infection. After the desired length was achieved in the LON group, the fixator was removed and two distal interlocking screws are inserted; partial weight bearing was continued until full consolidation. In the conventional group, the fixators were removed when the individual was fully weight bearing and after radiographic confirmation of 3 cortices in the regenerate column of both AP and lateral X-ray images.

We used the percentage increase in length (PI), external fixation index (EFI) and consolidation index (CI). PI is defined as the length gained divided by the original length. EFI is defined as the duration of external fixation divided by the length gained. CI is defined as the time of consolidation (from the operation day to the confirmation of consolidation) divided by the length gained. Consolidation was considered to be complete when anteroposterior and lateral radiographs confirmed at least three of four cortices were intact. We recorded the complication rate and the types of complications occurring within each group.

The independent Student’s *t* test was used to analyse the differences between the two groups. Differences in the number of complications were assessed with the Pearson chi-squared test. A *p* value of <0.05 was regarded as significant and a *p* value <0.001 was regard as statistically highly significant.

## Results

The mean duration of follow-up was 18 months (12–24 months).

There was a highly significant difference between the 2 groups in EFP and EFI and an insignificant difference in PI and CI. The results are shown in Table 3.

**Table 3:** A comparison between outcomes of the 2 groups

Indices	LON group	Conventional group	Significance (student’s *t* test)
Length gained (cm)	4 (1.8–9.1)	4.98 (3–8)	0.833
Percentage increase (%)	11.2 ± 6.2 %	13.96 ± 8.3 %	0.33
External fixation period (days)	52.2 (30–120)	180.4 (110–260)	<0.001
External fixation index (days/cm)	13.2 (10.32–16.66)	37.08 (32.5–46.25)	<0.001
Consolidation index (days/cm)	42.3 (31.28–58.18)	37.08 (32.5–46.25)	0.006

The complications were grouped according to Paley’s system [3]. The mean number of complications was 0.8 per limb in the LON group compared to a mean of 1.4 per limb in the conventional group (*P* < 0.001). Details of complications are shown in Table 4.

**Table 4:** Details of complications

	LON				Conventional			
	Problem	Obstacle	Sequelae	Total	Problem	Obstacle	Sequelae	Total
PTI	5	0	0	5	9	0	0	9
IMI	0	0	3	3	0	0	0	0
Axial deviation	0	0	0	0	3	2	0	5
Refracture	0	0	0	0	2	1	0	3
Joint contracture	0	1	0	1	3	3	0	6
Delayed consolidation	3	0	0		0	0	0	0
Total	8	1	3	12	17	6	0	23

*PTI* pin tract infection, *IMI* intramedullary infection

There were three cases of equinus contracture requiring tendo-Achilles lengthening; 1 in the LON group and 2 in the conventional group. Only 1 in the conventional group responded to physiotherapy alone. Two of the 3 cases of deep IMI (intramedullary infection) continued lengthening until consolidation. The infection was resolved after nail removal and reaming of the medulla. One case that was lengthened by the LRS rail fixator required premature nail removal, reaming and application of Ilizarov frame. All these patients showed no recurrence of infection for 12-month follow-up after nail removal.

## Discussion

The LON technique evolved to avoid complications of prolonged external fixation and fracture after frame removal. Numerous studies were published on the technique [4, 7, 8, 11–14] but only a few [9, 10, 15] have compared it with the conventional Ilizarov protocol. Alike previous studies, we achieved a highly significant reduction in the mean EFI in the LON group compared to the conventional group. However, the mean CI was significantly higher in the LON group; this contradicts with the results of Guo et al. [10], Watanabe et al. [14] and Park et al. [9] who showed insignificant difference between the 2 groups and Sun et al. [15] who showed significant decrease in CI in the LON group. We explained this by the extreme values of 2 cases of deep IMI that must have delayed regenerate consolidation. If these values are excluded, the difference in CI between the 2 groups is insignificant. Park et al. [9] and Sun et al. [15] did not record any deep IMI in their LON group and Watanabe [14] and Guo et al. [10] also reported no incidence of deep infection.

Despite strict adherence to the recommendations of Paley et al. [4] of avoiding contact between the pin and the nail, the incidence of deep infection in LON was 20 % (3 cases) compared with 0, 3, 5, 15,2.4, 0, 9.5 and 11 % previously reported by Guo et al. [10], Paley et al. [4], Silberg et al. [11], Simpson et al. [13], Kouaglou et al. [7], Watanabe et al. [14], Kim et al. [8] and Kristiansen [6], respectively. This high percentage of deep infection is due to the small number of cases. These 3 patients were heavy smokers and not compliant with pin care instructions.

The effect of reaming the medullary canal on the quality of regenerate remains an issue of unresolved debate. According to our results, in contradiction with previous literature [9, 10, 14], the CI in the LON group was increased, due largely to the influence of the 3 cases that had deep IMI. This incriminates infection rather than reaming in delaying consolidation. To confirm this, further study is needed to compare between reamed and unreamed nailing in the LON group.

The original technique of LON describes a distal vent to avoid fat embolism during reaming. We vent the medulla through drill holes of the planned osteotomy which is later completed after reaming. We believe this carries the advantage of keeping the reaming material in the vicinity of the osteotomy and thus enhancing consolidation and decreasing the CI in the LON group. This was not proven in our sample.

The drawbacks in this study are a small number of cases, lengthening of different limb segments and the use of lengthening devices in both groups. However, the study does indicate that early substitution of external fixation for internal fixation can bring benefits but needs to be balanced against potential risks of deep implant-related sepsis. Further studies should be directed to comparing LON with lengthening and then nailing (LTN) and plate after lengthening (PAL). The potential of lengthening over an antibiotic-coated nail may provide a solution to the problem of deep IMI.
